# Association of Altered Serum MicroRNAs with Perihematomal Edema after Acute Intracerebral Hemorrhage

**DOI:** 10.1371/journal.pone.0133783

**Published:** 2015-07-24

**Authors:** Ying Zhu, Jia-Lu Wang, Zhi-Yi He, Feng Jin, Ling Tang

**Affiliations:** Department of Neurology, The First Hospital of China Medical University, Shenyang, China; National University of Singapore, SINGAPORE

## Abstract

**Background and Purpose:**

Perihematomal edema (PHE) contributes to secondary brain damage and aggravates patient outcomes after intracerebral hemorrhage (ICH). MicroRNAs (miRNAs) are stable in circulation, and their unique expression profiles have fundamental roles in modulating vascular disease. The objective of this study was to test the hypothesis that altered miRNA levels are associated with PHE in ICH patients.

**Methods:**

Hematoma and PHE volumes of ICH patients were measured on admission and in follow-up computed tomography scans. Whole-genome miRNA profiles of ICH patients and healthy controls were determined using the Exiqon miRCURY LNA Array, and validated by quantitative reverse transcription-polymerase chain reaction (qRT-PCR). Bioinformatics analysis investigated dysregulated miRNA target genes and the signaling pathways involved.

**Results:**

We identified 55 miRNAs that were differentially expressed in ICH patients compared with normal controls, of which 54 were down-regulated and one was up-regulated. qRT-PCR confirmation showed decreases in miR-126 (0.63-fold), miR-146a (0.64-fold), miR-let-7a (0.50-fold), and miR-26a (0.54-fold) in ICH patients relative to controls. Serum miR-126, but not miR-146a, miR-let-7a or miR-26a, levels were significantly correlated with relative PHE volume on days 3–4 (*r* = −0.714; *P*<0.001) in patients with ICH.

**Conclusions:**

ICH patients appear to have a specific miRNA expression profile. Low expression of miR-126 was positively correlated with the extent of PHE, suggesting it may have a pathogenic role in the development of PHE after ICH.

## Introduction

Intracerebral hemorrhage (ICH) accounts for 10–15% of all strokes, and affects over a million people worldwide annually, most of whom either die or are left seriously disabled [1]. It is therefore a major public health problem, for which no treatment has yet proven effective. Various factors have been shown to influence the patient outcome in ICH, including age, volume of ICH, hematoma growth, neurologic deficit, presence of intraventricular blood, and infratentorial location [2–4]. Among these factors, hematoma growth usually occurs in the early phase of ICH, and is strongly associated with poor outcome. Based on the time course and proximity to the hematoma, perihematomal edema (PHE) is commonly observed during the acute and subacute stage, and it may be more accountable for the poor neurological outcome than the hematoma mass itself [5,6]. Therapeutic intervention aimed at attenuating the extent of PHE could therefore represent an acute treatment paradigm for ICH.

MicroRNAs (miRNAs) are a class of small (18–22 nucleotides) non-coding RNAs that have fundamental roles in the post-transcriptional regulation of gene expression [7]. Serum miRNAs are present in a stable form that is protected from endogenous RNase activity. Moreover, their expression level was shown to be consistent among individuals of the same species [8], which makes them useful biomarkers for disease diagnosis and might help to develop new therapeutic options. Recently, miRNAs have been implicated in the pathogenesis of various neurological and vascular diseases [9], while preliminary studies indicated that miRNAs are dysregulated in the blood of stroke patients. However, few published studies have focused on miRNAs and ICH, and some used animal models rather than humans [9–12]. Thus, information about the relationship between miRNAs expression in humans and the occurrence of PHE in ICH is limited. The identification of specific miRNA signatures in PHE may aid the diagnosis and risk stratification. Therefore, the present study compared miRNAs expression profiling changes in ICH patients and healthy controls to explore the impact of PHE on ICH.

## Materials and Methods

### Patients

We prospectively evaluated patients aged 18 years or older who presented with acute symptomatic and radiologically confirmed ICH. Patients were eligible for entry if they presented within 24 h of onset with a primary basal ganglia hemorrhage and absence of coma. They were excluded if they had undergone surgery, had a known secondary ICH (hemorrhage resulting from aneurysm, vascular malformation, hemorrhagic infarction, or while under anticoagulant treatment), or experienced an intraventricular hemorrhage. Patients were initially evaluated between May 2013 and June 2014 at the First Hospital of China Medical University (Shenyang, China), and healthy controls matched for age and sex were recruited at random from individuals undergoing a regular health check-up. The study has been approved by the ethics committee of The First Hospital of China Medical University and written informed consent was obtained from all participants. The approval number: [2012]38–1

### Routine clinical assessment

We recorded baseline demographic characteristics, medical history, vascular risk factors, clinical presentation, hemorrhage onset-to-imaging time, blood glucose levels and pressure on admission, neurological deficits (according to the National Institutes of Health stroke scale) at time of treatment, complications, presumed cause of ICH, and details of laboratory tests.

### Computed tomography (CT)

All patients underwent two cranial CT scans: an initial scan on admission (<24 h), and a second 3–4 days after the onset of symptoms (follow-up CT scan). All CT scans were performed according to standard techniques. Investigators who analyzed the images were blinded to clinical and biomarker information. All images were processed off-line with the use of imaging processing software running on an AW4.2 GE Advantage workstation. ICH and edema volumes were calculated using a semi-automated process.

**Fig 1:**
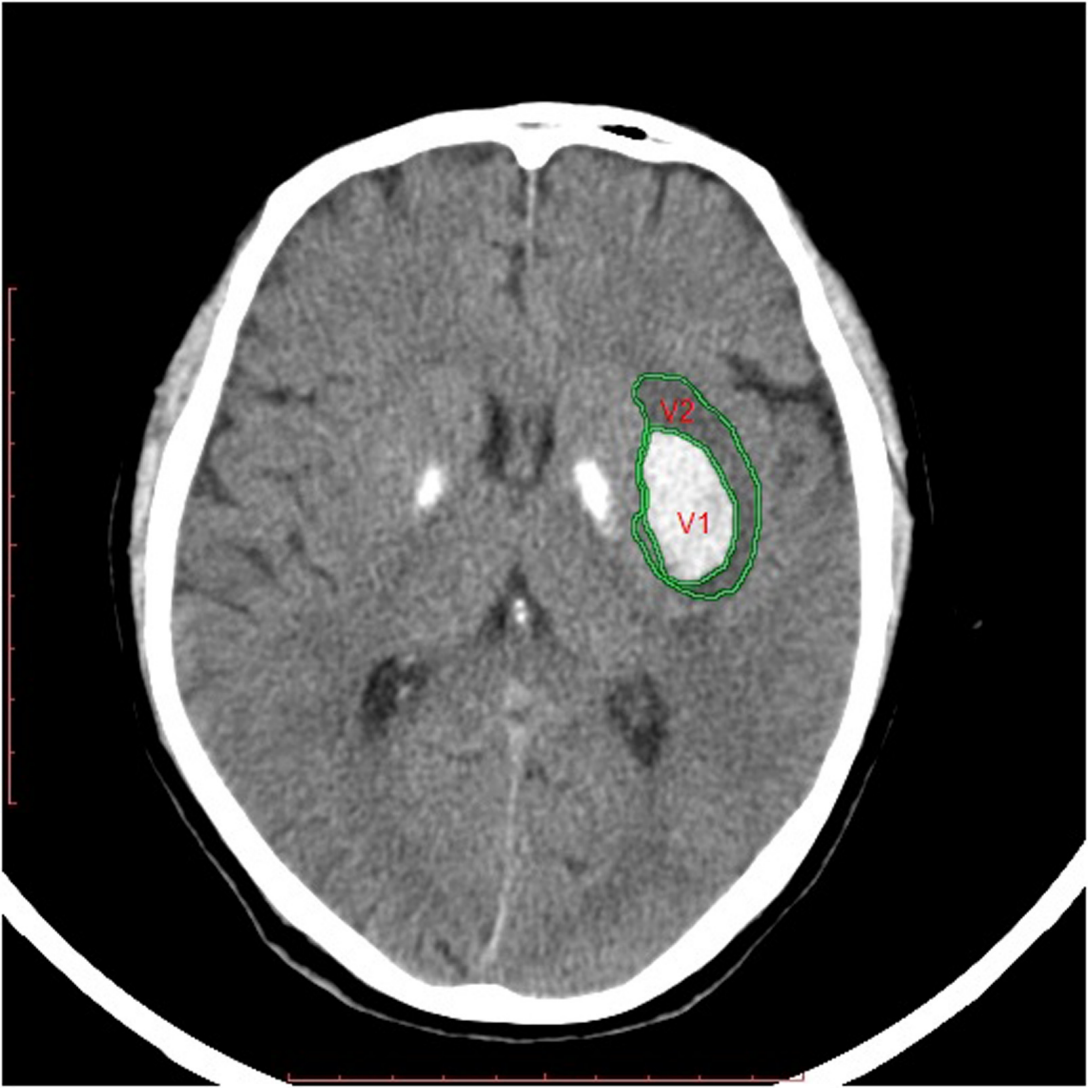
Depiction of the measurements of PHE and hematoma. (V1) Hyperdense hematoma regions and (V2) hypodense area surrounding hematoma for PHE. Volumes from all slices were summed: rPHE = V2/V1. PHE, perihematomal edema.

The examiner manually drew regions of interest by tracing the hyperdense perimeter representing hematoma (Hounsfield unit range, 40–100 HU), and the hypodense region surrounding the hematoma, indicating PHE (range, 5–33 HU), in each slice throughout the lesion. ICH and total lesion volumes were calculated by multiplying the specific traced area by slice thickness and summing the results (Fig 1). PHE was measured by subtracting the hyperdense volume (ICH area) from the total lesion area (hyperdense + hypodense lesion area). Relative (r)PHE was then calculated by dividing the absolute PHE by hematoma volume. When very small, unmeasurable amounts of edema were present, values of zero were assigned for both absolute and relative edema volume [13].

### Sample collection and processing

Blood samples were collected from controls at study entry and from patients on admission. Blood was centrifuged for 10 min, after which serum was aspirated, aliquoted in RNAse-free tubes, and stored at −80°C until required for further processing.

RNA was extracted from fixed volumes of serum using Trizol LS reagent following the manufacturer’s protocol. RNA concentration and purity were evaluated using a spectrophotometer. cDNA synthesis was performed using the Universal cDNA Synthesis Kit according to the manufacturer’s instructions. miRNA expression was examined using miRCURY LNA miRNA chips. The Exiqon arrays contained 384 probes specific for human mature miRNAs. Amplification was performed on the 7900HT thermocycler using cycling parameters recommended by Exiqon. The median of triplicate probes was used for each array, and expressed data were normalized using the median normalization method. Data analysis was performed using GenEx2.0 Software.

### Candidate miRNA confirmation and quantification by real-time quantitative PCR

Reverse transcription reaction products were combined with 2×SYBR Green master mix (Exiqon), and polymerase chain reaction (PCR) amplification was carried out in a final volume of 10 μl using a 7900 HT Real-Time PCR System (Applied Biosystems). PCR conditions were: 10 min incubation at 95°C followed by 40 cycles of 95°C for 15 s and 60°C for 1 min. Threshold cycle (CT) values >38 were defined as undetectable. miRNA levels were normalized to those of miR-191-5p as an internal control. The relative fold-change between ICH patients and control samples for each miRNA was calculated using the comparative Ct (2^-ΔΔCT^) method, and the results were assessed by a *t*-test.

### Statistical analysis

Data are expressed as means ± SD or medians (with interquartile ranges). Statistical analyses were performed using SPSS software version 19.0 (SPSS, Inc., Chicago, IL, USA). Statistical significance for intergroup differences was assessed by the χ^2^ test or Fisher’s exact test for categorical variables, and by the Student’s *t*-test, or Mann–Whitney *U* test for continuous variables. Correlations between variables were tested with Spearman’s correlation analysis. A *P*-value <0.05 was considered significant for all tests.

## Results

### Baseline characteristics

Demographic characteristics of the participants are shown in Table 1. A total of 33 participants with new-onset ICH and were recruited into this study (13 men and 20 women; median age, 57.37 ± 9.67 years) together with 15 healthy volunteers (eight men and seven women; median age, 58.4 ± 9.66 years). There was no significant difference in age or sex distribution between the ICH patients and healthy volunteers.

**Table 1:** The clinical features of ICH patients and normal controls.

Baseline characteristics	Controls(N = 15)	All patients(N = 33)	*P* values
Age,y,mean±SD	58.4±9.66	57.37±9.67	0.73
Male sex,n(%)	53.30%	37.10%	0.288
Hypertension,n(%)	33.30%	100%	<0.001
Diabetes mellitus,n(%)	36.40%	20%	0.602
Smoking history,n(%)	36.40%	28.60%	0.891
White cell count,10^9^/L, mean±SD	6.17±1.59	9.22±2.64	0.001
Platelet,10^9^/L, mean±SD	224±69	203±53	0.244
INR, s, mean±SD	1.07±0.1	1.01±0.1	0.053
Fibrinogen, g/L, mean±SD	3.03±0.67	3.46±0.76	0.063
LDL, mmol/L, mean±SD	2.92±0.5	3.07±1.03	0.598
Glucose, mmol/L, mean±SD	6.56±1.87	6.71±1.89	0.793
Baseline hematoma volume, ml, mean±SD	-	29.57±14.55	NA
Baseline PHE volume, ml, mean±SD	-	19.74±12.54	NA
Hematoma volume on day 3–4,ml,mean±SD	-	25.48±14.15	NA
PHE volume on day 3–4,ml,mean±SD	-	40.27±31.97	NA
Relative PHE on day 3–4,mean±SD	-	1.37±0.55	NA

ICH, intracerebral hemorrhage; SD, standard deviation; INR, international normalized ratio; LDL, low-density lipoprotein; PHE, Perihematomal edema; NA, not available.

### miRNA expression analysis reveals an altered profile in ICH patients

We first screened for differentially expressed serum miRNAs in six ICH patients compared with four healthy controls. ICH patients were found to have a serum miRNA profile that differed significantly from that of healthy controls. We identified a total of 55 differentially expressed miRNAs exhibiting fold-changes ≥1.5 (*P*<0.05) among patients and controls, of which 54 were down-regulated and one was up-regulated (S1 Table). Of these, miR-126 (0.39-fold), miR-146a (0.33-fold), miR-let-7a (0.46-fold), and miR-26a (0.31-fold) were notably decreased in the ICH group compared with the control group.

### qRT-PCR analysis of miRNA expression

**Fig 2:**
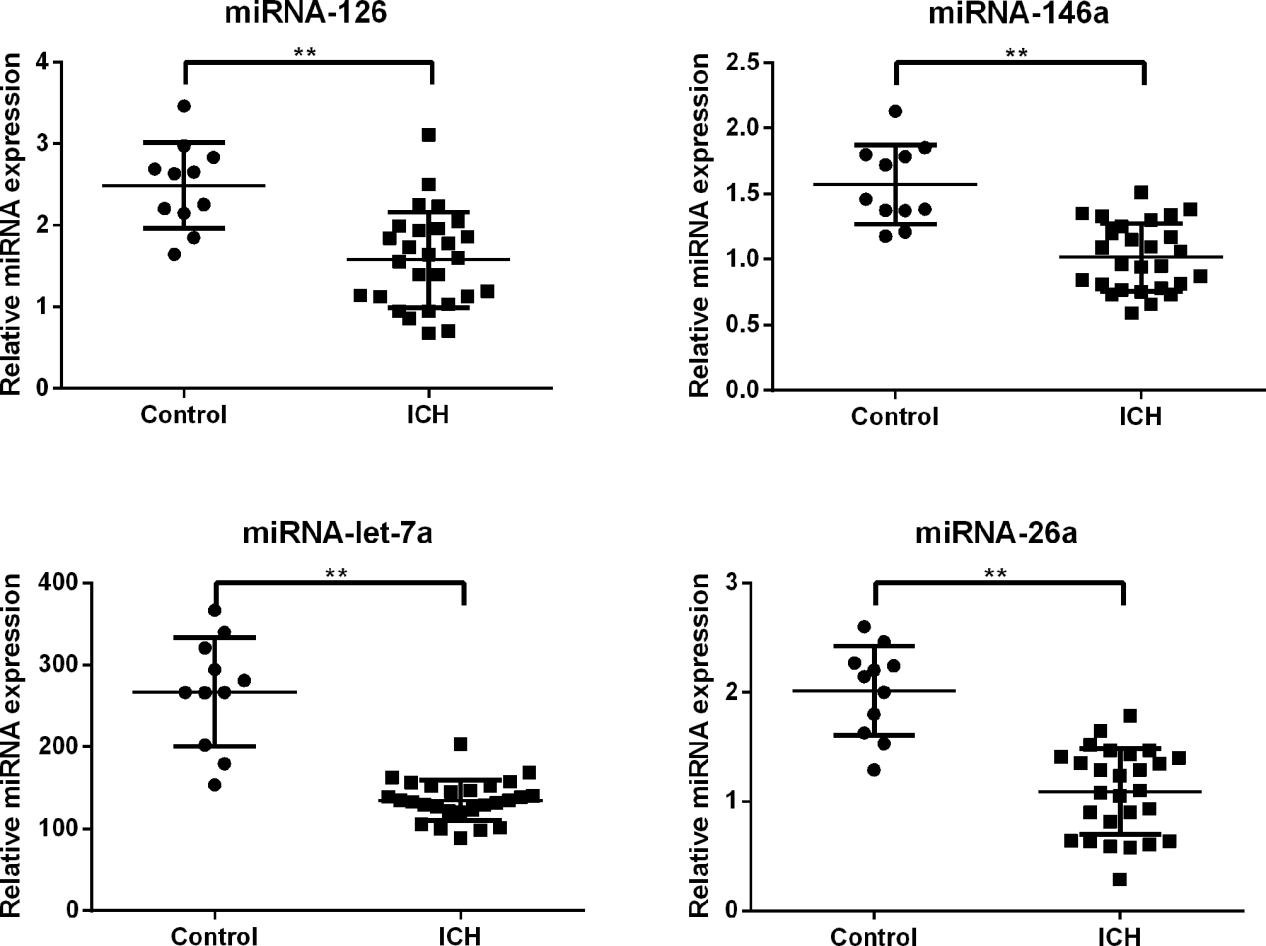
Four serum miRNA levels were selected for verification using real-time qRT-PCR in individual ICH patients (n = 27) and healthy controls (n = 11). Serum levels of miR-126, miR-146a, miR-let-7a, and miR-26a were significantly lower in ICH patients compared with those in the control group (**, *P*<0.01). miRNA expression levels are normalized to miR-191-5p (relative level). ICH, intracerebral hemorrhage.

We then selected four representative down-regulated miRNAs previously shown to be associated with ICH (miR-126, miR-146a, miR-let-7a, and miR-26a) with microarray *P* values < 0.01 for qRT-PCR validation. This confirmation study was carried out in 27 ICH patients and 11 controls. miR-126, miR-146a, miR-let-7a, and miR-26a were all present at significantly lower levels in patients relative to controls, with average fold-changes of 0.63, 0.64, 0.50, and 0.54, respectively(*P*<0.01) (Fig 2A–2D).

### Target gene prediction

To further investigate the possible function of the four miRNAs showing fold-changes, we performed bioinformatic data mining using the TargetScan algorithm to produce a target gene list for the differentially expressed miRNAs. Gene functional annotation analysis showed that some genes targeted by miR-126, miR-146a, miR-let-7a, and miR-26a are involved in PHE formation after ICH. The biological processes associated with these genes include the innate immune response, leukocyte activation (miR-126 and miR-146a), response to oxidative stress (miR-146a), programmed cell death (miR-let-7a), and smooth muscle cell proliferation (miR-26a). The miRNA entities under each category and their target genes relevant to ICH are summarized in Table 2.

**Table 2:** Functional classification of changed miRNAs in ICH and their target genes.

miRNA	target genes	Process or function categories involved
miR-126[20,32]	*VCAM1,SPRED1,PIK3R2*	Immune response
		Vascular function
miR-146a[23,24,27]	*SMAD4,TRAF6,CFH,IRAK-1,IL-6,IL-8*	Immune response
		Oxidative stress response
		TGF beta receptor pathway activity
miR-let-7a[30,33]	*TP53,CASP3,AKT2,HIF1AN,ACVR2A,IL6, IL10, FASLG, IGF1,TGFBR1*	Apoptosis
		Oxidative stress response
		Smooth muscle cell proliferation
		TGF beta receptor pathway activity
miR-26a[28,29]	*BAK1,PTEN,SMAD1,SMAD4,KLF4,IGF1*	Smooth muscle cell proliferation
		TGF beta receptor pathway activity
		Oxidative stress response
		Apoptosis

ICH, intracerebral hemorrhage.

### Association of miR-126, miR-146a, miR-let-7a, and miR-26a levels with the extent of PHE in ICH

**Fig 3:**
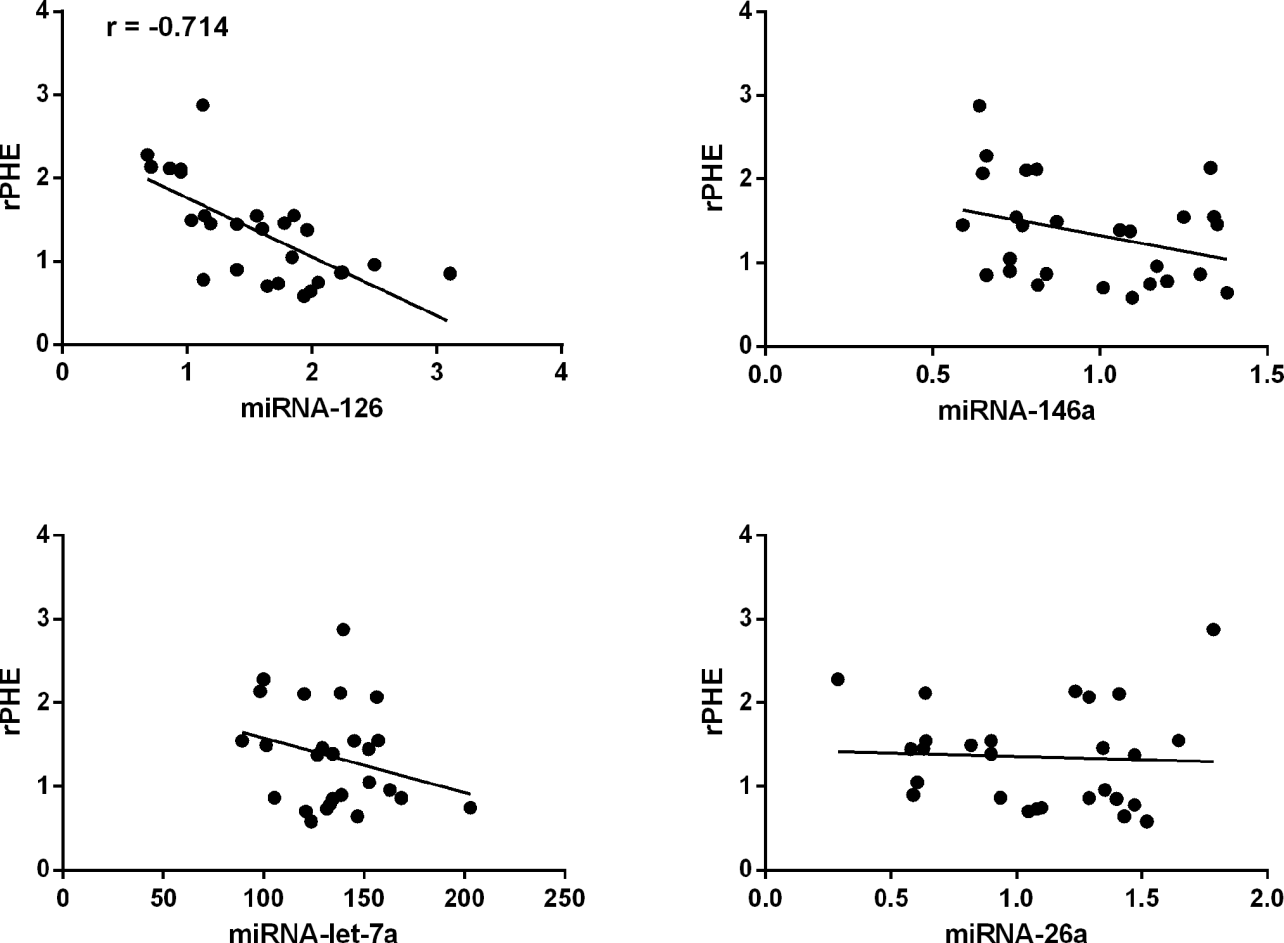
Spearman correlation analysis of serum miR-126, miR-146a, miR-let-7a, and miR-26a levels with rPHE in samples from ICH patients (n = 27). Note that serum levels of (A) miR-126, but not (B–D) miR-146a, miR-let-7a, or miR-26a, were significantly correlated with rPHE. rPHE, relative perihematomal edema.

To investigate whether these altered miRNAs are relevant to PHE development, we used the Spearman’s rank correlation coefficient to determine the presence or absence of a correlation between these variables. Only the concentration of serum miR-126 was significantly correlated with rPHE (r = −0.714; *P*<0.001; Fig 3A). By contrast, there was no significant correlation between serum miR-146a, miR-let-7a, or miR-26a concentrations and rPHE (Fig 3B–3D). Spearman’s correlation analysis was also performed between miR-126 levels and other clinical parameters of the ICH patients, but no correlations were observed (Table 3).

**Table 3:** Spearman correlation analysis between the level of serum miR-126 and the clinical characteristics of the ICH patients.

Variable		miR-126
Age	R	-0.044
	P value	0.828
Sex	R	0.288
	P value	0.146
Diabetes mellitus	R	-0.134
	P value	0.506
SBP	R	0.047
	P value	0.816
White cell count	R	-0.265
	P value	0.182
Fibrinogen	R	-0.153
	P value	0.445
LDL	R	0.005
	P value	0.982
Glucose	R	-0.286
	P value	0.148

ICH, intracerebral hemorrhage; SBP, systolic blood pressure; LDL, low-density lipoprotein; R, Spearman correlation coefficient.

## Discussion

In this study, we compared miRNA expression profiles in ICH patients with those of healthy controls and evaluated the association between serum miRNA levels and rPHE volume. In a recent study, Guo et al [10] reported that 30 miRNAs were significantly up-regulated in ICH, of which most were inflammatory cell-derived miRNAs. This contrasts with our own study that identified only one up-regulated miRNA in ICH, but 54 that were down-regulated, while no miRNA down-regulation was detected previously. This discrepancy may reflect the different times at which blood samples were taken, or the small sample size.

Among the altered miRNAs in our own study, four (miR-126, miR-146a, miR-let-7a, and miR-26a) were confirmed to be significantly decreased in ICH patients by qRT-PCR, and miR-126 was positively correlated with rPHE volume in this cohort. Although changes in miRNA levels have previously been reported to be associated with ICH [10,11], to the best of our knowledge, this is the first study to analyze the association between serum miRNA levels with rPHE volume in patients with acute ICH. Additional well-designed prospective studies are needed to fully investigate our provisional findings.

Serum miRNAs have been widely used for clinical diagnosis and prognosis. In a previous study of brain and blood miRNA expression profiling in rat models, Liu et al. [12] reported different patterns of miRNA expression 24 h after brain ischemia, brain hemorrhage, and kainite-induced seizures compared with control animals, indicating the possible use of circulating miRNAs as biomarkers for one or more specific types of brain injuries. Although we could not directly assess miRNA levels in the vicinity of hemorrhagic brain tissue, serum miRNAs might nevertheless indicate the availability of miRNAs in the brain area of ICH.

The pathogenesis of PHE in ICH is unclear. In recent years, rapid progress has been made in basic molecular immunology and molecular biology, and our understanding of the cytokines involved in PHE formation has improved. It is now thought that a complex inflammatory response involving cellular (leukocytes, macrophages, and microglia) and molecular (cytokines, proteases, and reactive oxygen species) components rapidly occurs in ICH-induced brain edema [14–17], although the exact mechanisms have not been fully elucidated.

Previous studies have shown that serum miRNAs can influence the dysregulation of inflammatory cytokines and chemokines, and are associated with a proinflammatory state that can contribute to a number of pathologic changes [15,18]. miR-126 is highly enriched in endothelial cells, and promotes angiogenesis and inhibits inflammation by repressing the three target genes Sprouty-related EVH1 domain-containing protein 1, phosphoinositol-3 kinase regulatory subunit 2, and vascular cell adhesion molecule 1 (*VCAM-1*). VCAM-1 is an important adhesion molecule that contributes to immune cell localization at the site of inflammation. miR-126 knockdown up-regulates VCAM-1 expression, while miR-126 up-regulation during the acute injury phase inhibits VCAM-1 expression, thereby reducing the number of immune cells infiltrating the site of inflammation, which may attenuate the progression of PHE [12, 19–21].

PHE reduction could be an important adjunctive therapeutic target to improve the outcome of ICH patients, in contrast to hematoma expansion that often occurs within the first few hours of onset [22]. The delayed time window of miRNA-mediated injury may facilitate treatment. If prediction of the extent of PHE could be made based on miR-126 serum levels, this would prove useful in choosing treatment for ICH such as the administration of dehydration agents or surgery. Further investigations to explore the underlying mechanisms might lead to the development of treatment for the prevention of secondary brain damage and other unfavorable outcomes.

Bioinformatic analysis and previous studies revealed that miR-146a, miR-let-7a, and miR-26a are associated with several biological processes linked with the development of ICH-induced brain edema including the innate immune response, leukocyte activation, transforming growth factor-β signaling, and smooth muscle cell proliferation [12,15,18, 23–30]. However, neither miR-146a, miR-let-7a, nor miR-26a were associated with rPHE in our study. This could reflect the different time points of sample assessment and small number of patients compared with other studies.

Our investigation has a number of limitations. First, although it was a prospective study, the inclusion of patients was not strictly consecutive, so some selection bias may have occurred. For instance, it is possible that patients with severe neurological deficits at admission (i.e., with large hematomas and coma) or those who underwent a surgical evacuation were excluded, which would underestimate the occurrence of PHE in our series. Second, miRNA gene targets were identified using target databases. Because we did not perform experiments to modulate miRNA levels, our findings are indirect and the exact pathological role of the miRNAs remains unknown. Additional studies are therefore required to experimentally evaluate miRNA gene targets and to determine their functional role in ICH. Furthermore, we only investigated miRNA levels over 3–4 days, yet significant changes in ICH have been reported over time [31]. Further investigations conducted in larger numbers of patients and healthy volunteers are therefore required to validate our findings.

In summary, our data show that there were extensive changes in miRNA expression in ICH patients. In particular, miR-126 expression levels positively correlate with rPHE, indicating its potential usefulness as a biomarker for PHE management. A full understanding of the target genes of miR-126, as well as of the molecular mechanisms of the miRNA linked to PHE, may help elucidate the pathogenesis of PHE and further the clinical applications of miRNA-based therapy.

## Supporting Information

S1 Table MiRNAs that are differentially expressed in ICH patients as compared to healthy controls. (DOC)
