# Supplementary material for: Association of Altered Serum MicroRNAs with Perihematomal Edema after Acute Intracerebral Hemorrhage
Source: PLoS One. 2015 Jul 24;10(7):e0133783. doi: 10.1371/journal.pone.0133783 (PMC4514469; doi:10.1371/journal.pone.0133783)
Supplement: S1 Table — (DOC) [file pone.0133783.s001.doc]

S1 Table. MiRNAs that are differentially expressed in ICH patients as compared to healthy controls.

| miRNA ID | Fold Change | *P* value | Regulation |
| --- | --- | --- | --- |
| hsa-miR-27a-3p | 0.02 | 0.0133 | down |
| hsa-miR-205-5p | 0.04 | 0.0007 | down |
| hsa-miR-151a-3p | 0.10 | 0.0140 | down |
| hsa-miR-375 | 0.14 | 0.0093 | down |
| hsa-miR-605 | 0.14 | 0.0031 | down |
| hsa-miR-424-5p | 0.17 | 0.0011 | down |
| hsa-miR-195-5p | 0.17 | 0.0022 | down |
| hsa-let-7e-5p | 0.19 | 0.0005 | down |
| hsa-miR-146b-5p | 0.20 | 0.0993 | down |
| hsa-miR-99a-5p | 0.20 | 0.0177 | down |
| hsa-miR-145-5p | 0.22 | 0.0087 | down |
| hsa-miR-10a-5p | 0.23 | 0.0328 | down |
| hsa-let-7c | 0.23 | 0.0057 | down |
| hsa-miR-95 | 0.24 | 0.0231 | down |
| hsa-let-7f-5p | 0.25 | 0.0133 | down |
| hsa-miR-141-3p | 0.25 | 0.0006 | down |
| hsa-miR-30e-3p | 0.25 | 0.0089 | down |
| hsa-miR-107 | 0.26 | 0.0084 | down |
| hsa-miR-142-3p | 0.26 | 0.0099 | down |
| hsa-miR-15a-5p | 0.27 | 0.0159 | down |
| hsa-miR-99b-5p | 0.27 | 0.0243 | down |
| hsa-miR-30d-5p | 0.27 | 0.0189 | down |
| hsa-miR-28-5p | 0.28 | 0.0188 | down |
| hsa-miR-766-3p | 0.28 | 0.0296 | down |
| hsa-miR-23b-3p | 0.30 | 0.0310 | down |
| hsa-miR-335-5p | 0.30 | 0.0285 | down |
| hsa-miR-26a-5p | 0.31 | 0.0018 | down |
| hsa-miR-146a-5p | 0.33 | 0.0110 | down |
| hsa-miR-374b-5p | 0.34 | 0.0003 | down |
| hsa-miR-103a-3p | 0.34 | 0.0062 | down |
| hsa-miR-10b-5p | 0.34 | 0.0029 | down |
| hsa-miR-30a-5p | 0.36 | 0.0426 | down |
| hsa-miR-584-5p | 0.37 | 0.0089 | down |
| hsa-miR-30e-5p | 0.38 | 0.0341 | down |
| hsa-miR-151a-5p | 0.39 | 0.0115 | down |
| hsa-miR-126-3p | 0.39 | 0.0236 | down |
| hsa-miR-155-5p | 0.40 | 0.0109 | down |
| hsa-miR-30c-5p | 0.40 | 0.0094 | down |
| hsa-miR-21-5p | 0.40 | 0.0438 | down |
| hsa-miR-29b-3p | 0.41 | 0.0410 | down |
| hsa-miR-374a-5p | 0.42 | 0.0336 | down |
| hsa-miR-28-3p | 0.43 | 0.0233 | down |
| hsa-let-7d-5p | 0.44 | 0.0085 | down |
| hsa-miR-23a-3p | 0.44 | 0.0186 | down |
| hsa-let-7a-5p | 0.46 | 0.0374 | down |
| hsa-miR-223-5p | 0.46 | 0.0197 | down |
| hsa-miR-150-5p | 0.47 | 0.0465 | down |
| hsa-miR-106b-3p | 0.48 | 0.0182 | down |
| hsa-miR-221-3p | 0.49 | 0.0955 | down |
| hsa-miR-342-3p | 0.50 | 0.0307 | down |
| hsa-miR-425-3p | 0.50 | 0.0472 | down |
| hsa-miR-421 | 0.51 | 0.0116 | down |
| hsa-miR-30b-5p | 0.52 | 0.0499 | down |
| hsa-miR-191-5p | 0.57 | 0.0316 | down |
| hsa-miR-296-5p | 9.77 | 0.0209 | up |
